# Improving Biosensors by the Use of Different Nanomaterials: Case Study with Microcystins as Target Analytes

**DOI:** 10.3390/bios11120525

**Published:** 2021-12-20

**Authors:** Hanbin Park, Gahyeon Kim, Yoseph Seo, Yejin Yoon, Junhong Min, Chulhwan Park, Taek Lee

**Affiliations:** 1 Department of Chemical Engineering, Kwangwoon University, Seoul 01897, Korea; binwlal@naver.com (H.P.); 1497rg@hanmail.net (G.K.); akdldytpq12@gmail.com (Y.S.); wjwj0131@naver.com (Y.Y.); 2 School of Integrative Engineering, Chung-Ang University, Seoul 06974, Korea

**Keywords:** microcystin, nanoparticle, biosensor, cyanobacterial harmful algal bloom

## Abstract

The eutrophication of lakes and rivers without adequate rainfall leads to excessive growth of cyanobacterial harmful algal blooms (CyanoHABs) that produce toxicants, green tides, and unpleasant odors. The rapid growth of CyanoHABs owing to global warming, climate change, and the development of rainforests and dams without considering the environmental concern towards lakes and rivers is a serious issue. Humans and livestock consuming the toxicant-contaminated water that originated from CyanoHABs suffer severe health problems. Among the various toxicants produced by CyanoHABs, microcystins (MCs) are the most harmful. Excess accumulation of MC within living organisms can result in liver failure and hepatocirrhosis, eventually leading to death. Therefore, it is essential to precisely detect MCs in water samples. To date, the liquid chromatography–mass spectrometry (LC–MS) and enzyme-linked immunosorbent assay (ELISA) have been the standard methods for the detection of MC and provide precise results with high reliability. However, these methods require heavy instruments and complicated operation steps that could hamper the portability and field-readiness of the detection system. Therefore, in order for this goal to be achieved, the biosensor has been attracted to a powerful alternative for MC detection. Thus far, several types of MC biosensor have been proposed to detect MC in freshwater sample. The introduction of material is a useful option in order to improve the biosensor performance and construct new types of biosensors. Introducing nanomaterials to the biosensor interface provides new phenomena or enhances the sensitivity. In recent times, different types of nanomaterials, such as metallic, carbon-based, and transition metal dichalcogenide-based nanomaterials, have been developed and used to fabricate biosensors for MC detection. This study reviews the recent advancements in different nanomaterial-based MC biosensors.

## 1. Introduction

Cyanobacterial harmful algal blooms (CyanoHABs) are toxic algal blooms that float on living organisms, freshwater systems, and water supply sources during the summer and produce toxicants [1,2]. The eutrophication of lakes and rivers without adequate rainfall leads to excessive growth of CyanoHABs that produce toxicants, green tides, and unpleasant odors. The massive growth of CyanoHABs owing to global climate change and global warming has led to rapid eutrophication in water bodies [3,4,5].

The development of rainforests without considering the environment and construction of dams in rivers could result in the overgrowth of CyanoHABs [6]. Additionally, the industrialization of rural areas located near rivers and lakes is accelerating the growth of CyanoHABs, which can cause serious economic, health, and ecological problems [7]. Furthermore, humans, livestock, and aquatic animals could suffer from damage to the liver and other organs by drinking large amounts of CyanoHAB-contaminated water, leading to death [8,9]. Moreover, due to the growing amounts of CyanoHABs, the water bodies turn green and produce a stench smell, which is not esthetically pleasing. Particularly during the summer, the overgrowth of CyanoHABs produces algal blooms in waterbodies such as rivers, lakes, and ponds [10].

CyanoHABs produce harmful cyanotoxins such as microcystin, anatoxin, saxitoxin, nodularin, and cylindrospermopsin [11,12]. When CyanoHABs undergo damage or die, they release cyanotoxins that cause hepatotoxicity and neural toxicity to humans, livestock, and other wildlife [13,14]. Microcystin, nodularin, and cylindrospermopsin are classified as hepatotoxins, whereas anatoxin-a and saxitoxin are classified as neurotoxins [15,16].

Microcystins (MCs) are produced by various cyanobacteria genera, such as Microcystis, Planktothrix, Anabaena, Nostoc, Aphanizomenon, and Limnothrix, and are the most commonly found toxins from CyanoHABs [17]. MCs comprises seven amino acids having circular forms, and over 90 different types of MCs have been reported worldwide [18]. MC was classified with amino acid composition such as MC-LR, MC-RR, MC-YR, MC-LR, and MC-LF. These different MC species showed the different toxicities. It is reported that MC-LC showed the highest toxicity in comparison to the other MC types [19]. When MCs are exposed to living organisms, they cause inhibition of protein phosphatase in liver cells, protein kinase activation malfunction, and over-phosphorylation of proteins, thereby resulting in several acute diseases [20]. Furthermore, excess accumulation of MC in liver cells results in apoptosis of cells due to cytoskeletal disruption and control loss of p53 gene regulation. The structural stability of MC is determined in cells that do not decompose for 2–3 months [21]. Therefore, the precise detection of MC in freshwater is essential for humans and wildlife.

Liquid chromatography–mass spectrometry (LC–MS) and enzyme-linked immunosorbent assay (ELISA) are two of the conventional methods used for the detection of MC [5,19,22,23,24]. The United States Environmental Protection Agency (USEPA) recommends using these techniques to quantify the MC and nodularins in water samples as official methodologies “Method 546” and “Method 544” [23,24,25]. While LC–MS provides precise data and results, it requires heavy analytical apparatus and expensive equipment. Comparatively, while ELISA does not require heavy and expensive equipment, it does require a complicated detection step and trained researchers with extensive analytical time. Therefore, these techniques cannot meet the requirements of simple field-ready detection methods.

In the meantime, biosensors can detect various molecules, including toxins, and can be used as an alternative to solve the above problems. Several biosensors have been devised to detect various analytes such as viruses, pathogens, diseases, toxicants, and microorganisms [26,27,28,29,30]. The biosensors are designed to meet specific goals according to various detection platforms such as electrochemical [26], electrical [31], optical [32], and spectroscopic platforms [33]. The biosensor can provide the useful platform with small size, portability and easy-to-handle nature, and field-ready measurement [34]. In constructing a biosensor, the target recognition bioreceptors should be required. Generally, two types of bioreceptors are used to bind with specific targets. The antibody is a gold standard for immunosensor construction. It provides specific target binding, low Kd constant and high selectivity. However, the manufacturing cost of antibody is expensive, and production of antibody requires animal experiments. In last 20 years, the aptamer was regarded as a powerful alternative for biosensor construction. Aptamers are short RNA or DNA strands that can bind with high specificity and affinity to target materials such as proteins, lipids, ions, and whole cells by forming a unique 3D structure by folding. Aptamers are inexpensive, easy to synthesize, and small in size, exhibiting excellent chemical stability [35]. In addition, owing to their unique structural properties, aptamers can reportedly be enhanced through systematic evolution of ligands by exponential enrichment (SELEX) to significantly improve sensitivity and selectivity when binding to target substances. Compared to antibodies, it can be chemically synthesized, reducing the manufacturing cost and being free from animal experiments. However, the aptamer showed less selectivity and stability that should be solved by aptamer-based biosensor (aptasensor) commercialization.

Various types of biosensors can detect the toxicant small molecule effectively [36,37,38]. In particular, several reviews on biosensors for microcystin detection have been published [39,40,41]. Cunha et al. discussed the aptasensor for aquatic phycotoxins and cyanotoxins [39]. In this review, they mainly focused of application of aptamer for toxin detection. In addition, Bertani and Lu recently introduced the cyanobacterial toxin biosensors for environmental monitoring and protection [40]. They introduced various cyanotoxin biosensors, including saxitoxin, microcystin, and cylindrospermopsin. Moreover, this review focused on the portability of biosensor for field-ready application. Massey et al. recently summarized the microcystin detection methods [41]. They focused not on biosensors but on ELISA and HPLC-based MC detection. Thus, these reviews discussed MC detection methods using biosensor or conventional methods; however, these reviews did not explain the usefulness of introduction of nanomaterial for MC biosensor construction.

Meanwhile, the introduction of nanomaterial for the construction of biosensors provides detection sensitivity and selectivity, as well as new detection platforms [42,43].

Several nanomaterials have been synthesized for application in fields of energy, medicine, science, and engineering [44,45,46,47]. Among them, three types of nanomaterials: noble metal-based, carbon-based, and transition metal dichalcogenide (TMD)-based nanomaterial, are useful for the construction of toxicant-detecting biosensors [48,49,50]. A suitable platform for detecting CyanoHABs and algal toxins can be achieved by combining adequate bioreceptors (antibody, aptamer, and nucleic acid) with the above-mentioned nanomaterials. The present review discusses the recent progress in the four types of nanoparticles and bioreceptors hybrid material-based MC biosensors.

## 2. Metal Nanoparticle-Based MC Biosensor

Metal-based nanoparticles with various physical and chemical properties according to their composition and shape have also been developed [48,49,50,51]. Metal nanoparticles are widely used in batteries, materials, devices, and for the treatment of cancer [52,53]. Furthermore, noble metal nanoparticles such as gold, silver, and rhodium nanoparticles exhibit superior conductivity and high stability and are used in electrochemical biosensors for the detection of microcystin [48,54,55].

Owing to the electrical or electrochemical properties of conductive nanoparticles, they can be used in MC biosensors to improve the detection sensitivity of the sensor by increasing the surface area of the interface between the target and the bioreceptors electrode. Furthermore, conductive nanoparticles can be used in electrochemical and electricity-based biosensors by pairing them with an antibody or an aptamer to achieve high detection sensitivity.

**Figure 1:**
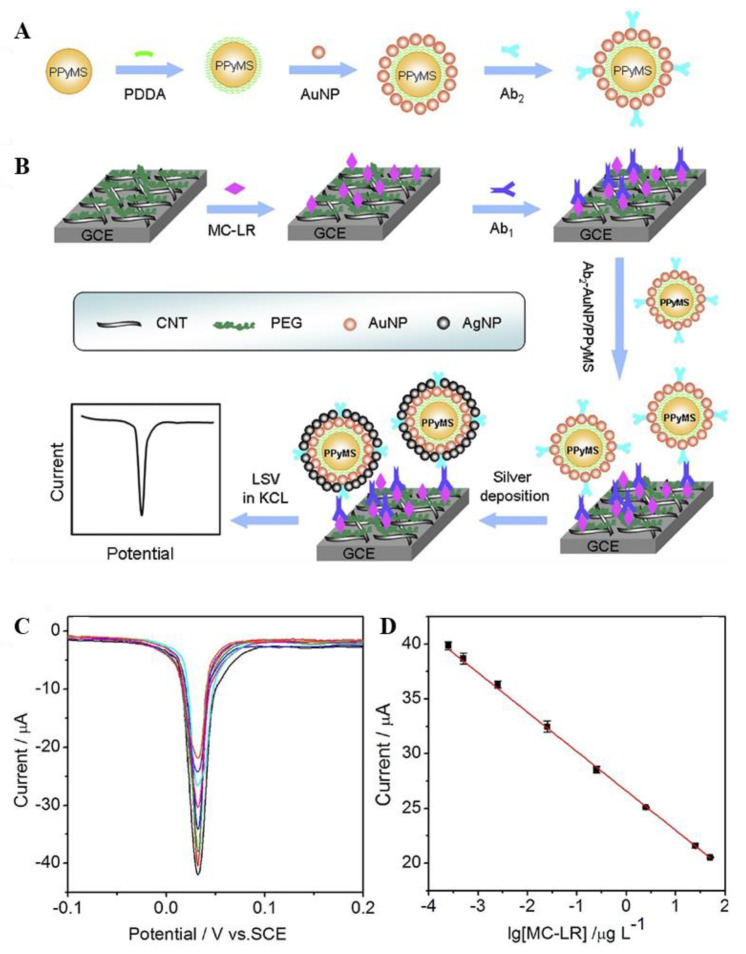
(**A**) Ab2-AuNP/PPyMS particle preparation method. (**B**) Schematic representation of the MC-LR immunosensor fabrication and competition immunoassay procedure. (**C**) Linear sweep stripping voltammetry curves of silver nanoparticles deposited on the immunosensors at 1.0 M KCl after incubation with the lowest peak currents in the MCLR having corresponding concentration ranges (0.00025, 0.0005, 0.0025, 0.025, 0.25, 2.5, 25, and 50 μg/L). (**D**) Calibration curve for MC-LR immunoassay. Reproduced with permission from [56], published by Elsevier, 2017.

Electrochemical measurement type can be applied to immunosensor for MC-LR detection with high selectivity and sensitivity. The analytical approach exhibits acceptable precision, stability, and accuracy. Zhang et al. [56] developed electrochemical immunosensors using functional *PPy* microspheres (AuNP/PpyMS) comprising gold nanoparticles for the detection of microcystin-LR. The fabricated biosensor was composed of antibody–AuNP/PPyMS complex for generating electrochemical signal enhancement through AuNP. AuNP was prepared by depositing silver on polypyrrole microspheres that were synthesized by chemical oxidation polymerization to act as electrochemical catalysts for signal amplification. As shown in Figure 1A, AuNPs were incorporated into polypyrrole microspheres as signal antibodies, and the detection signal was enhanced by increasing the surface area. Furthermore, the electrodes of the MC-LR immunosensor were modified using carbon nanotubes (CNTs), and the excellent fixation of MC-LR antigens to the modified electrodes was due to the stabilization of the antigen binding site of the polyethylene glycol (PEG) film. Moreover, the coated MC-LRs were subjected to MC-LR antibodies to form antigen–antibody complexes for competitive immunolysis, which reacted with the AuNP/PPyMS-labeled signal antibodies to generate electrochemical signals under the silver catalyst.

The detection performance of MC-LR was evaluated using linear sweep voltammetry (LSV) peak currents, wherein the current of the working electrode is measured, while the potential between the working and reference electrodes changes linearly with time. Figure 1B shows the LSV peak current measured in the concentration range of 0.0005–100 μg/L, which could be used for immunoanalysis, owing to the tendency of the current to decrease as the concentration of MC-LR antigens used in peak competitive immune responses increased. The antigen–antibody immune complex reacts with Ab2-AuNP/PPyMS and induces reduction of silver ions in the dissociated Cl^−^ in KCl. Although the linearization curve (Figure 1C,D) of MC-LR immunolysis had a low detection limit of 0.2 ng/L in the corresponding linear range, the proposed analytical approach showed excellent stability and high precision, exhibiting potential applications for the detection of other toxins.

Secondly, noble metal nanomaterials arising from nanoscale phenomena such as localized surface plasmon resonance (LSPR) or enhancement of Raman signals are also used in the fabrication of MC biosensors. The vibrations occur at the surface of the metal nanoparticles, which have localized surfaces, in the surface plasmon resonance (SPR) sensor. This results in LSPR referred to as the resonance formed by combining the electromagnetic field (EM) with spatially limited free electrons [57]. LSPR can be excited resonantly around nanoparticles of metal surfaces or thin films of metal. Furthermore, the wavelength intensity and length of LSPR can be altered by combining the bioreceptors and target material on the surface of the nanoparticles, which was confirmed by the LSPR results [58]. Moreover, LSPR-based biosensors have a simple structure, are easy to operate, and are portable for detection in the field [59].

One of the most essential characteristics of metal nanoparticles such as Au and Ag is that LSPR is generated in metal nanoparticles. The optical plasmonic properties of metal NPs are highly dependent on the grain boundary distance between the NPs, which are small or large aggregates of NP pairs, compared to individual and well-spaced NPs. As the interparticle distance decreased, a strong overlap was observed between the plasmon fields of the surrounding particles, owing to which the intensity increased, causing a redshift in the LSPR band, making it easier to observe changes in the color solution. AuNPs and AgNPs exhibit excellent LSPR properties with strong and distinct colors and color changes between individual NPs. Compared to aggregated NPs, widely spaced NPs are easier with UV-visible spectroscopy. This can be visualized or confirmed [60].

**Figure 2:**
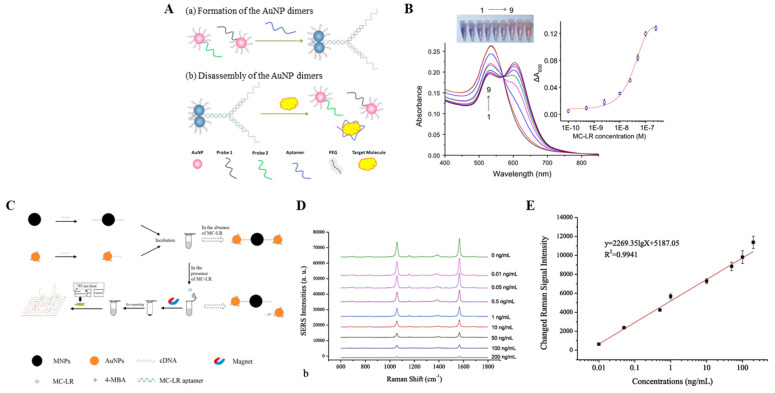
(**A**) Schematic representation of the formation and disassembly of AuNP dimers. (**B**) Extinction spectra of the sensors for different concentrations (0, 0.1, 0.5, 2.5, 10, 25, 50, 100, and 250 nM) of MC-LR. (**C**) Illustration of the principle behind the MC-LR-based analysis on the SERS-based aptasensor. (**D**) Typical SERS spectra with exposure to different concentrations of MC-LR (0–200 ng/mL). (**E**) Linear correlation between the changed Raman signal intensities and concentrations of MC-LR. Reproduced with permission from [61,68], published by Elsevier, 2015 and 2019, respectively.

Wang et al. fabricated an LSPR-based immune sensor using AuNP–aptamer assays for MC-LR detection [61]. As shown in Figure 2A, a target molecule-specific aptamer was used as the linker to prepare the AuNP dimer. Then, the AuNP dimer was degraded in the presence of the target molecule, and the color of the solution changed from blue to red. Furthermore, a new peak appeared at approximately 606 nm, and the absorbance increased at 606 nm as the linker concentration increased (Figure 2B). Conversely, in the absence of the target molecule, the aptamer acts as a linker that induces the formation of an asymmetrically altered AuNPs dimer, owing to which the solution appears blue, thereby indicating that the absorption peak of the AuNP monomer was at approximately 539 nm, similar to that before the linker was added. Compared to omnidirectional sensors based on the expansion of large aggregates into molecules, LSPR-based sensors exhibit significantly high sensitivity and stability and can be measured within a duration of 5 min.

Raman spectroscopy is used as a molecular identification tool, considering the fact that it enables the qualitative and quantitative analysis of molecules by measuring the vibrational spectrum of the sensor. Raman scattering mainly depends on the energy loss (Stokes) or gain (anti-Stokes) of inelastically scattered photons, owing to the molecular vibrational events, and reflects information about the molecular structure to enable in situ real-time sensing [62,63]. However, because the signal strength of the spectrum sensor was weak, surface-enhanced Raman spectroscopy (SERS) using specific metals was developed [64,65]. In SERS, the Raman signal of a chemical target material is amplified by the resonance between the wavelength of the incident light and the surface free electrons form when the chemical target material is close to a specific metal nanosurface. The SERS mechanism can be divided into electromagnetic and chemical enhancements. In general, the contribution of chemical enhancement is smaller than that of electromagnetic enhancement. SERS is advantageous, given that unlabeled non-destructive analytes can be detected from the spectral results, such as fingerprints and individual components of biochemical molecules and multi-component materials, respectively [66,67].

In Deyun’s study, MC-LR was detected with high sensitivity using the SERS technology-based AuNP-aptasensor [68]. The overall schematic of the MC-LR detection method is the same as that shown in Figure 2C. The MC-LR aptamer and its corresponding complementary DNA fragment (cDNA) bind the gold (AuNPs) and magnetic (MNPs) nanoparticles, respectively. Furthermore, MC-LR aptamer–AuNPs and cDNA–MNP conjugates were used as signal reporter and bioreceptors, respectively. Figure 2D,E shows linearity ranging between 0.01 and 200 ng/mL with a proposed sensor detection limit (LOD) of 0.002 ng/mL. The reliability of the new approach was evaluated at different concentrations of spiked MC-LR in tap water samples.

Li et al. fabricated a sandwich SERS spectroscopic immunosensor comprising a surface-functionalized quartz substrate and a SERS tag to selectively detect MC-LR [69]. The SERS tag utilized the gold nanospike (GNS) plasmonic substrate, owing to its SERS augmentation factor, unique high-density “hotspots,” and easy tuning of the LSPR band to the near-infrared (NIR) region. The GNS was identified at 750 nm using a 785 nm NIR laser excitation. NTP molecules used as Raman reporters were tightly adsorbed and immobilized on the GNS surface, which resulted in the GNS @ NTP @ SiO_2_ structure. Herein, we demonstrate how the developed SERS sensor can reach a detection limit of 0.14 µg/L. A high-performing SERS immunosensor assay allows for monitoring of the dynamic generation of MC-LR.

A colorimetric sensor that uses the degree of aggregation of conductive metal nanoparticles is also an interesting approach. Metallic nanoparticles are known to have attributes such as controllable physical and chemical properties, functional flexibility, low toxicity, and excellent stability, making them ideal sensor materials [70]. Colorimetric sensors have attracted wide attention in biochemical analysis, owing to their simplicity, high sensitivity, and low cost [71]. Among the different metals, gold (AuNPs) has been used in various colorimetric sensors, owing to its wide visibility of color change [72]. However, metal nanoparticles as sensing elements can sometimes lack the ability to selectively target specific materials. Therefore, to solve this problem, recent colorimetric biosensors research has developed a nanoparticle–aptamer complex where nanoparticles are bound to the aptamer, which is an oligonucleotide with excellent selectivity for a target material used as the sensor material [73,74,75,76].

However, to date, existing analytical techniques used for the detection of ML-LR in fresh water have many limitations, such as the interference of various environmental organic pollutants, specialized technology, complex sample preparation, expensive equipment, and lengthy detection time. Therefore, to overcome these problems, recent colorimetric sensor studies have suggested using Au nanoparticle–aptamer-based colorimetric sensors, which are simple and highly sensitive, specifically for MC-LR detection [77,78,79].

**Figure 3:**
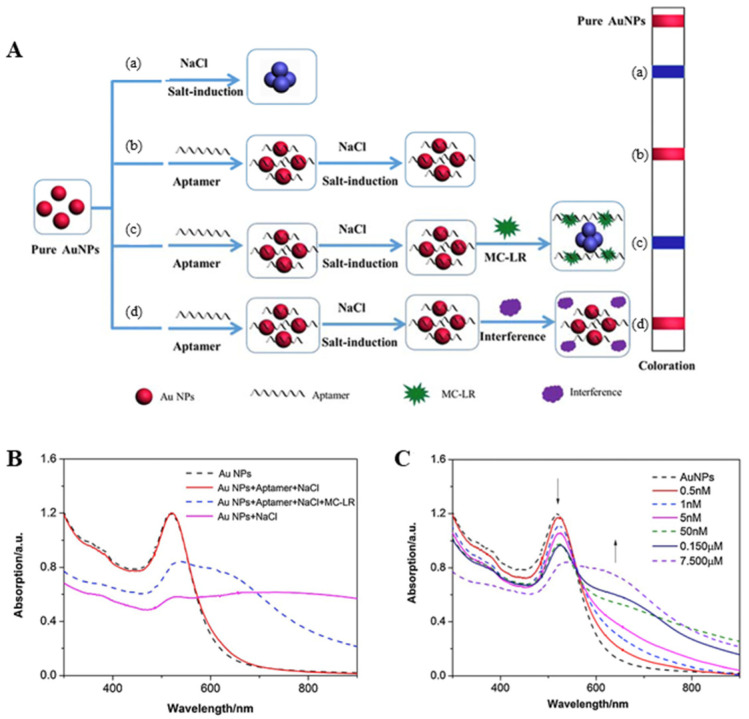
(**A**) Schematic illustration of the colorimetric sensor and mechanism of analytic determination of MC-LR. (**B**) UV–VIS absorption spectra of AuNPs under different experimental conditions, c(Au NPs) = 4.4 nM, c(aptamer) = 0.128 μM, c(NaCl) = 24 mM, c(MC-LR) = 750 nM, T = 298K.C. (**C**) UV–VIS absorption spectrum of Au NPs (concentration range of MC-LR: 0.5 nM–7.5 μM). Reproduced with permission from [77], published by Elsevier, 2016.

The Au nanoparticle–aptamer-based colorimetric sensor principle used in the study by Li et al. (2016) [77] is described as follows. The aptamer and metal nanoparticles are used as recognition elements to selectively bind to MC-LR with high affinity and as sensing materials to detect the color change in the plasma resonance absorption peak when binding to a target in high-concentration sodium chloride, respectively (Figure 3A–C). When the Au nanoparticle–aptamer comes in contact with the target material (MC-LR) in the sample, the aptamer structure modifies and separates from the nanoparticles to form an MC-LR/aptamer complex. Thereafter, a concentrated salt solution was used to aggregate the released AuNPs, and the sensor was driven by a method that changes the color of the sample by interparticle plasmon coupling of metal nanoparticles. The limit of detection (LOD) of the MC-LR-specific AuNP–aptamer was estimated as 0.37 nM. In addition, it was observed for the real sample that the biosensor worked even in high-salt pond water. Therefore, given that this colorimetric sensor has low cost, excellent stability, and reproducibility and can determine the presence of MC-LR even in real freshwater, it is considered very useful for detecting MC-LR in the real environment.

## 3. Carbon Nanomaterial-Based MC Biosensor

Owing to the unique structural characteristics of carbon, since 2000, carbon-based nanomaterials such as carbon nanotubes, carbon nanofibers, graphene, and graphene oxide have been used for the development of electrochemical, electrical, and spectroscopic biosensors [80,81,82,83]. Recently, a new type of structural layer called MXene was developed, which improved the scalability of the applicability of biosensors [52,84]. Recent studies have attempted using carbon-based biosensors with excellent sensor performance for MC detection.

**Figure 4:**
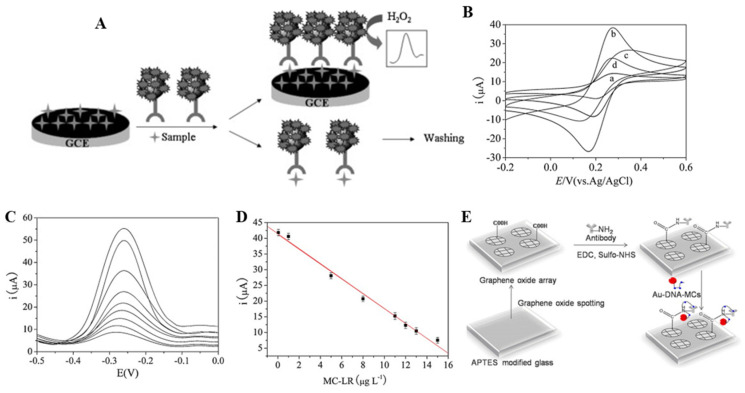
(**A**) Schematic illustration of the detection principles of the immunosensor using microcystins–LR/graphene sheets–chitosan/GCE and horseradish peroxidase–carbon nanosphere–antibody bioconjugates. (**B**) Cyclic voltammograms of (a) Bare GCE, (b) GSs-CS/GCE, (c) MC-LR/GSs-CS/GCE, (d) HRP-CNSs-Ab/MC-LR/GSs-CS/GCE, (e) in 0.1 M KCl containing 2.5 mM Fe(CN)_6_^3^^−^/Fe(CN)_6_^4^^−^ mixture (1:1 molar ratio). Scan rate: 60 mV s^−^^1^. (**C**) In 0.2 M PBS (pH 7.4), DPV measurement results at MC-LR concentration shifts (0.05 to 15 μg/L). The current response of the immunosensor after incubation with amplitude: 50 mV, pulse width (top to bottom) containing 7.0 mM H_2_O_2_. The DPV measurements were performed from −0.5 V–0 V, with an amplitude of 50 mV and a pulse width of 0.01 s. (**D**) The calibration curve of the current responses vs. MC-LR concentrations and liner fit for microcystin-LR concentrations. (**E**) Illustration of GO-based fluorescence biosensor. Reproduced with permission from [85,95] published by Elsevier, 2013 and 2012.

Zhao et al. [85] developed graphene and multienzyme functions as carbon-nanosphere-based electrochemical immune sensors for MC-LR detection. The immune sensors used graphene (GSs) and chitosan (CS) to improve the electrochemical performance of the electrodes. Furthermore, a horseradish peroxidase–carbon nanosphere (CNS)–antibody system was used to amplify the electrochemical signal. Figure 4A shows a mimetic diagram of an electrochemical immune sensor comprising bio-composite nanostructures horseradish peroxidase–carbon nanosphere–antibody and microcystins–LR/graphene sheets–chitosan/glassy carbon electrode (GCE).

The above-mentioned sensor was formed by the immobilization of nanocomposite GSs-CS/CNS to the vitreous carbon electrode. The detection sensitivity was improved by using a synthesized HRP-CNS-Ab bio junction. The sensor has a three-dimensional structure that can be used as a signal reporter and interacts with MC-LR. Thereafter, hydrogen peroxide was applied to transfer electrons directly from the electrode to form an electrical signal. Owing to the electrode, the formation of free-state HRP-CNS-Ab/MC-LR/GSs-CS/GCE decreased as the concentration of the MC-LR samples increased.

Figure 4B shows the measurements of redox peaks using the cyclic voltammetry (CV) method, a method of obtaining the current potential curve from a solid electrode at each stationary stage of the sensor. The periodic changes to the electrode potential were obtained using a triangular wave [Fe(CN)_6_] ^3−/4−^ as the redox species. The applied voltages were measured, ranging between −0.2 and 0.6 V. CV was obtained when the GCE (curve b) electrodes were modified into GS-CS, thereby resulting in a 38% increase in current of the over bare GCE (curve b) electrodes, indicating that the electrodes modified with GS-CS exhibited increased conductivity and a larger surface area. However, the MC-LR/GSs-CS/GCE (curve c) showed a decrease of 30.8% in the current value from curve b, which indicated that the MC-LR was fixed to the electrode surface. This reduction can be attributed to the inhibition of the electron transfer process by the MC-LR, which is nonconductive. The insulation properties of the electrode surface increased further after incubation with HRP-CNS-Ab (curve d), thereby reducing the peak current response.

Figure 4C,D shows the results of the evaluation of the sensor performance using the DPV measurement method. A linear range of 0.05–15 μg/L microcystin-LR with a detection limit of 0.016 μg/L was observed. Furthermore, the DPV measurements under optimized conditions (buffer: 0.2 M PBS (pH7.4), applied voltage: −0.5–0 V, amplitude: 50 mV pulse width: 0.01 s) were proportional to the concentration of MC-LR and decreased linearly as the MC-LR concentration increased in the 0.05–15 μg/L concentration range. The linear range obtained for MC-LR was much wider than the range of immune sensors (0.06–0.65 μg/L) derived from antibodies labeled directly with HRP.

In addition, various studies have attempted to detect MC-LR using carbon materials that exhibit excellent conductivity. For example, Zhang et al. (2011) [86] fabricated an immunosensor by coating AuNPs onto nitrogen-doped carbon nanotubes. Furthermore, Zhao et al. (2013) [85] fabricated a graphene-based immunosensor using a horseradish peroxidase–carbon nanosphere–antibody system for signal amplification to detect microcystine-LR.

Among the various carbon materials, graphene separated from crystalline graphite is one of the representative biosensor materials. Graphene is an allotrope of carbon comprising a single atom-thick and a flat monolayer comprising a two-dimensional sheet of honeycomb lattice [87], having unique optical, electronic, thermal, and chemical properties. Owing to this, graphene and its derivatives are emerging as the new carbon materials and are attracting wide attention in different fields such as biological detection [88], nanocomposite synthesis [89], and microelectronic device fabrication [90]. However, considering the limitations of existing physical approaches [91], the chemical modification and functionalization of graphene has attracted attention from many researchers. In particular, graphene oxide (GO) prepared by oxidizing graphite has abundant hydrophilic groups (hydroxyl, epoxide, carboxyl groups, etc.) on its surface, which indicates that it can be well dispersed in water [92,93]. Furthermore, GO maintains a delocalized π–electron system that provides a strong affinity to the carbon-based ring structure of graphene, being known to have superior value as a sensor material compared to graphene [94].

The work of Shi et al. [95] introduced GO to biosensors in their study (Figure 4E). The sensor substrate was constructed by exposing a coated glass slide surface to APTES vapor. Graphene oxide was adsorbed through electrostatic force using a graphene oxide array. Here, crosslinking agents EDC and sulfo-NHS were added to activate the exposed carboxyl group of GO through incubation. Furthermore, a carboxyl–amine group covalent bond was formed on the graphene oxide surface through additional incubation after adding an NH2-MC antibody solution. In the detection step, the AuNP–ssDNA complex was formed with MCs in the sample by poly (A) ssDNA (5′-AAA AAA AAA AAA AAA-3′), and the residual complex, which was not bound to the MCs, was eliminated by poly (T) ssDNA (5′-TTT TTT TTT TTT TTT-3′). The bound MCs were recognized immunologically by the antibodies (NH2-MCs) adsorbed onto the GO surface. The GO and AuNPs act as–donor–acceptor pairs to induce quenching of GO fluorescence through the FRET phenomenon. Therefore, when the AuNP–ssDNA–MC complex is formed in GO, MCs are detected using a mechanism that lowers the intensity of fluorescence.

The limit of detections of MC-LR and MC-RR in the sensor were 0.5 mg/L and 0.3 mg/L, respectively, which satisfied the strictest standards of the World Health Organization (WHO). Furthermore, we obtained significant fluorescence quenching signals from MCs in real lake water. The antibody was able to recognize the Adda ((all-S, all-E)-3-amino-9-methoxy-2,6,8-trimethyl-10-phenyldeca-4,6-dienoic acid) group in the MC structure, which is a conservative part of MCs, to sensitively and selectively detect MCs. Results showed that the GO-based sensor exhibits high sensitivity, acceptable stability, and high reproducibility, thereby indicating the possibility of using GO-based sensors for the detection of MC-LR in environmental samples.

## 4. Transition Metal Dichalcogenides Nanoparticle-Based MC Biosensor

Because the first paper describing the properties of transition metal dichalcogenides (TMDs) was published almost a decade ago, one-dimensional nanomaterials have become one of the most vibrant areas of study in the field of materials science [96,97]. Two-dimensional TMD nanostructures such as molybdenum disulfide (MoS_2_) and bismuth selenide (Bi_2_Se_3_) exhibit remarkable optical, electrical, magnetic, and mechanical properties and have attracted significant attention, owing to their potential applications in silicon-based devices and various material applications [98,99,100,101]. In particular, MoS_2_ has been studied extensively for the storage and conversion of electrochemical energy in the form of electrocatalyst for hydrogen evolution reactions, electrode material in lithium-ion batteries, and supercapacitors, owing to its good anti-corrosion, catalytic abilities, and biosensor [102,103,104]. Owing to these interesting characteristics, several groups have reported the application of MoS_2_ for the development of biosensors [105,106,107].

Liu et al. [108] developed a sensitive aptasensor based on a dual-signal amplification system that uses both horseradish peroxidase (HRP) and a trilobe nanocomposite (AuNP@MoS_2_-TiONB nanocomposite) for the measurement of MC-LR. MoS_2_ nanosheets that can cover spherical TiO_2_ surfaces have a large surface area, which increases the chances of binding to biomolecules. The HRP also amplifies the sensing signal of the sensor. The DPV method was used to evaluate the analytical performance of MC-LR under scanning conditions from 0.22 to 0.23 V at a pulse amplitude of 50 mV every 0.1 s. The peak current value decreased as the concentration of MC-LR increased in the concentration range of 0.005–200 nM, which can be attributed to the decrease in the binding of biotin–cDNA, which reduced the binding of avidin–HRP. Therefore, the electrocatalyst current of HRP was confirmed to be inversely proportional to MC-LR.

**Figure 5:**
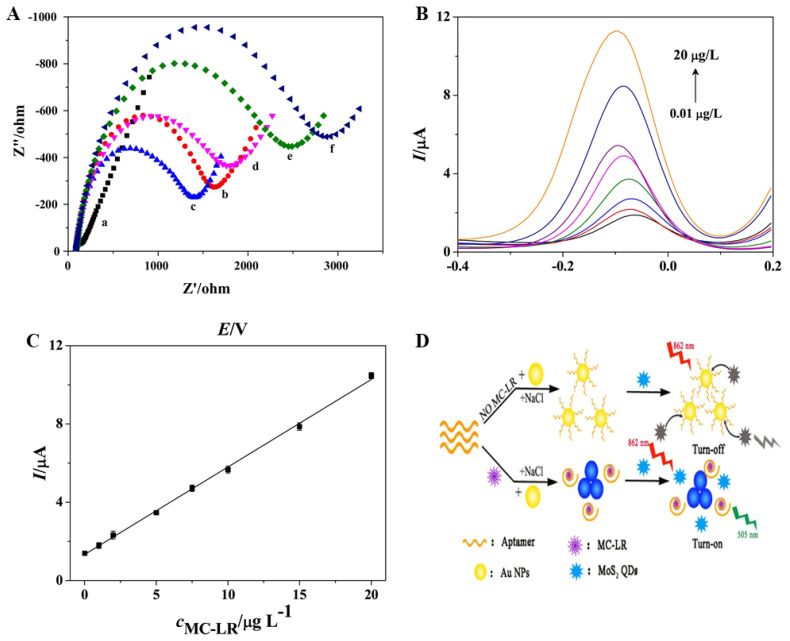
(**A**) Nyquist plots of (**a**) bare Au electrode, (**b**) MoS2/Au, (**c**) AuNRs/MoS2/Au, (**d**) anti-MC-LR/AuNRs/MoS2/Au, (**e**) MC-LR/anti-MC-LR/AuNRs/MoS2/Au, and (**f**) HRP–anti-MC-LR/MC-LR/anti-MC-LR/AuNRs/MoS2/Au in 20 mM PBS (pH 7.5, 0.10 M KCl) containing 5.0 mM [Fe(CN)_6_] ^3^^−^^/4^^−^ and 0.10 M NaClO4 within frequency range of 0.1 Hz to 100 kHz, amplitude of 0.05 V, and applied potential of 0.20 V. (**B**) DPV responses of the immunosensor in 20 mM PBS (pH 7.5, 100 mM NaClO_4_ and 100 mM KCl) containing 0.8 mM HQ and 2.0 mM H_2_O_2_ after incubation with (0.01 ~ 20.0 μg L^−^^1^) MC-LR. (**C**) Calibration curve for MC-LR immunoassay. The DPV measurements were performed from −0.4 V to 0.2 V, with an amplitude of 50 mV and a pulse width of 50 ms. (**D**) Schematic representation of the detection strategy of MC-LR according to MoS_2_ QDs using up conversion fluorescence and aptamer–AuNPs. Reproduced with permission from [109] published by Elsevier, 2017, and [113] published by American Chemical Society, 2020.

Zhang et al. [109] developed a sensitive electrochemical immunosensor based on molybdenum disulfide (MoS_2_) and gold nanorod (AuNR) composites for the detection of MC-LR. The immunosensor was constructed by immobilizing an MC-LR antibody on a gold electrode, which was modified using a MoS_2_/AuNRs nanocomposite with a large surface area and excellent biocompatibility. The detection method used had a competitive immunoassay format, wherein the coated MC-LR antibody competed with the added target MC-LR for the MC-LR antigen to form an antibody–antigen immunocomplex. Then, horseradish peroxidase-labeled anti-MC-LR antibody (HRP-Ab_2_) was used to detect MC-LR. Figure 5A shows a 20 mM PBS (pH 7.5, 0.1 M KCl) buffer containing 5 mM [Fe(CN)_6_] ^3−/4−^ and 0.1 M KClO_4_ in the frequency range of 0.1 Hz to 100 kHz at a bias potential of 0.2 V. The Nyquist plots, a diagram comprising a high-frequency semicircle and low-frequency linear section corresponding to the electron transfer resistance (*R_e_*_t_), of electrochemical impedance spectroscopy (EIS) for the electrodes were plotted at each modification step. It can be seen that, unlike other electrodes, the bare electrode (curve a) has a low electron transfer resistance (*R_et_*), indicated by a straight line. After modifying the AuNR in MoS_2_/Au (curve c), the *R_e_*_t_ value decreased, indicating that AuNR promoted [Fe(CN)_6_] ^3−/4−^ ion transport. In the other process, it was confirmed that the *R_e_*_t_ value increased, which indicated that MoS_2_, AuNR, anti-MC-LR, MC-LR, and HRP–anti-MC-LR were successfully immobilized on the surface of the gold electrode. The DPV measurement method was used to evaluate the electrochemical performance of the MC-LR measurement immune sensor. Under optimal conditions, the immunosensor showed a linear response to MC-LR in the range of 0.01–20 μg/L with a detection limit of 5 ng (Figure 5B,C). This electrochemical immune sensor showed excellent potential for monitoring routine water quality for various toxins.

A MoS_2_–quantum dot (MoS_2_ QD) complex system was synthesized and applied to a fluorescent biosensor as a fluorophore and a quencher, respectively. MoS_2_ QDs exhibit excellent stability, low toxicity, and low manufacturing costs. Furthermore, it has strong resonant light absorption, excellent photoluminescence, and excellent potential as a fluorometric sensor material [110,111]. Moreover, when N-acetyl-L-cysteine (NAC) is used as a capping agent in the synthesis of MoS_2_ QDs, in near-infrared absorption caused by NAC, abnormal upconversion photoluminescence of MoS_2_ QDs occurs, owing to the two successive energy transfers into the hexagonal MoS_2_ QD structure, which appears as green fluorescence under UV and NIR irradiation [112]. This upconversion photoluminescence by MoS–QDs under low-energy NIR irradiation is known to effectively eliminate background interferences [113].

On the basis of the unique luminescence of MoS_2_ QDs, Cao et al. (2020) [113] presented a method for detecting MC-LR according to MoS_2_ QDs using upconversion fluorescence generated by NAC. Furthermore, this method used MoS_2_ QDs as a signal-sensing molecule and gold nanoparticle–aptamer as a recognition factor for a target material (Figure 5D). The working principle of this study is similar to that of the colorimetric sensor proposed by Li et al. (2016) [77]. However, MoS_2_ QDs cannot bind to aggregated AuNPs in the post-separation process of AuNP–aptamer in the presence of MC-LR. Therefore, the sensor measures the upconversion fluorescence from the exposed MoS_2_ QDs. This method has a lower limit of detection (0.01 nM) as compared to a fluorescent sensor that uses a fluorophore and a quencher. In addition, the ability to eliminate background noise in complex environmental samples by emitting visible photoluminescence of MoS_2_ QDs with upconversion fluorescence indicates that the MoS– QD-based fluorescence sensor is highly effective in accurately detecting the presence of MC-LR in the environment.

## 5. MC Biosensors with Other Nanomaterials

**Figure 6:**
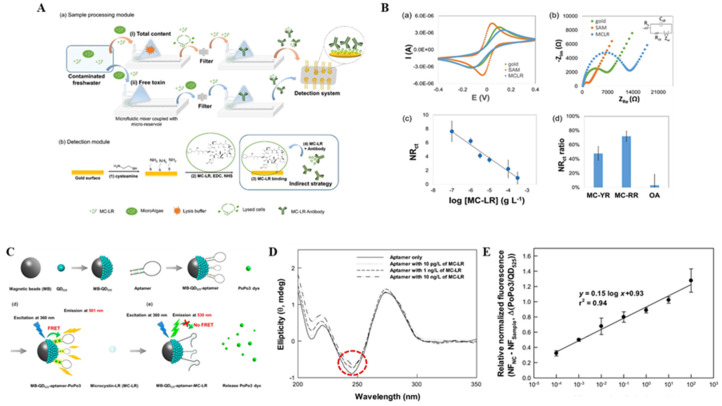
(**A**) Schematic representation of the overall MC-LR detection system. A sample processing system comprising (**a**) sample processing and (**b**) detection modules. Consists of (i) two microfluidic mixers coupled and (ii) reservoirs (μFM-μR) used to process freshwater cyanobacterial samples without lysis buffer. (**b**) Immunosensor fabrication; (1) diagram of SAM with cysteamine, (2) MC-LR activation, and (3) MC-LR binding to a SAM-modified surface. (**B**) Nyquist plots for (**a**) cyclic voltammetry and (**b**) surface functionalization steps; (inset) Randles equivalent circuit model used for EIS fitting. (**c**) Normalized signal of R_ct_ versus the MC-LR concentration (concentration of antibody 10^−2^ g/L). (**d**) Percentage of NRct signals obtained for the same concentration (10^−4^ g/L) of different toxins (MC-YR, MC-RR, OA). (**C**) Schematic representation of the MC-LR detection procedure of the FRET-based QD–aptasensor. (**D**) Circular dichroism (CD) spectral changes of the aptamer with MC-LR. (**E**) Sensitivity validation for MC-LR detection after 6 h of reaction. Reproduced with permission from [114,115], published by Elsevier, 2019.

For the fabrication of field-ready biosensors, it is essential to pretreat the cyanobacterial sample and detect MC simultaneously. Dos et al. [114] developed a good example of this system by developing a portable microfluidic sensing platform for simultaneously detecting MC-LRs in and out of Microcystis aeruginosa cells. The filter in the chip filtered the MC toxins that discriminated the sample and quantitatively detected MCs. Figure 6A shows the scheme of the manufactured sensor, wherein the sample processing module uses a micro-tank (μR), microfluidic mixer (μFM), and ultra-filter methods for pretreating the samples. The detection principle and functionalization of monetary poles follows electrochemical impedance spectroscopy (EIS) using an electrochemical cell chip (ECC). In-sample toxins competitively inhibit the binding of anti-MC-LR antibodies to the electrode surface where MC-LR is immobilized. The analytical performance was tested by characterizing the surface functionalization of ECC by increasing the concentration of MC-LR. Therefore, this process simultaneously detects the total MC-LR content (in and out of the cells) and concentration of MC-LR toxin (out of cells) in a water-free state.

Figure 6B shows the result of the performance analysis at each stage of electrode fabrication using CV and EIS methods. The surface functionalization stage CV measurements showed that the Faraday current of CV increase due to the immobilization of cysteamine in gold electrodes, which can be attributed to the electrostatic attraction between the positively charged monolayer (surface pK_a_ = 6.7) and negatively charged solutions, while the toxin bonded to the surface. The Nyquist plot was suitable for Randles equivalent circuit models. Furthermore, the formation of cysteamine SAM in gold electrodes reduced the R_ct_ value (1825 ± 306), whereas the combination of MC-LR on the SAM gold surface resulted in a higher R_ct_ signal (17,007 ± 4161) from MC-LR compared to SAM. The EIS measurements for MC-LR quantification were performed on custom electrochemical impedance portable platforms (EPP) (under conditions of 0.5–100,000 Hz, sinusoidal perturbation: 0.005 V). As a result, the N R_ct_ signal showed an increase in the sample concentration in the MC-LR concentration range (3.3 × 10^−4^–10^−4^ g/L). In addition, the similarity (MC-YR and MC-RR) between MC-LR with a concentration of 10^−4^ g/L at (d) and the NR_ct_ signals at OA verified the detection selectivity of the sensor.

In addition, various types of nanoparticles were introduced to fabricate the MC biosensor. Among them, core-shell structured nanoparticles and upconversion nanoparticles (UCNPs) were applied to MC biosensors. Lee et al. developed a highly sensitive fluorescence resonance energy transfer (FRET)-based quantum dot (QD)–aptasensor for the detection of MC-LR during the budding phase [115] (Figure 6C). Figure 6D shows the UV–VIS measurement results. A difference was visible at 245 nm, which can be attributed to the decrease in the intensity of the negative circular dichroism (CD) band when the target molecule is inserted perpendicular to the helical DNA axis, thereby indicating that the peak at approximately 245 nm is the only negative CD band and the intensity of that peak. It is assumed that the MC-LR molecule is inserted perpendicular to the base pair and binds to the back of the aptamer. The fret-based QD-aptasensor had a measured detection limit of 10^−4^ μg/L in the range of 10^−4^ to 10^2^ μg/L (ppb or nmol/L) (Figure 6E). MC-LR is selectively detected in different homologues of MC-LR such as microcystin-YR, microcystin-LY, microcystin-LW, microcystin-RR, microcystin-LF, microcystin-LA, and nojularin. Furthermore, the laboratory culture resulted in changes in the intracellular MC-LR concentration along the bacterial growth curve. In the early stages of fixation, QD–Aputasensa detected MC-LR in bacterial cultures of 12.7–15.8 μg/L. For environmental samples, MC-LR corresponded to 1.0 and 7.2 μg of MC-LR/L-water measured at 2.7 × 10^8^ and 6.6 × 10^10^ cells/L-water, respectively, indicating that microcystin-LR can be quantified in both laboratory cultures and eutrophic conditions [115].

Wu et al. developed an MC-LR sensor that uses green and red UCNP luminescence as donors and two quenchers, black hole quencher-1 (BHQ1), and black hole quencher-3 (BHQ3-). The two donor–acceptor pairs were constructed by hybridizing the aptamer with the corresponding complementary DNA. Results showed that the overlapping spectrum of green and red UCNP emissions can be extinguished by the bioreceptor. Aptamers preferentially bind to the corresponding analyte in the presence of MC-LR and okadaic acid (OA) and dehybridize with complementary DNA. The detection limits for MC-LR and OA were found to be 0.025 and 0.05 ng/mL, respectively. Experiments showed that the relative luminescence intensity increased as the algal toxin concentration increases, which promoted the quantification of MC-LR and OA [116].

## 6. Future Perspectives

Although developed countries are attempting to control the growth of cyanobacteria by limiting the construction of lake areas and improving the operation of water purification systems, developing countries are unable to ensure such control, and the sparse development is further accelerated. Furthermore, the indiscriminate increase in cyanobacteria owing to global warming is expected to increase in the future. The WHO has set a concentration value of 1 μg/L or less as the standard concentration of cyanobacteria in drinking water and recommends countries to comply with this. However, because this situation is relatively well observed in developed countries, there is still a long way to go. Therefore, the development of low-cost and highly reliable biosensors for the detection of MCs is expected to increase enormously.

Electrochemical and optical devices satisfy the above requirements in terms of ease of use, portability, and detection sensitivity. In particular, nanobiotechnology has led to the development of various sensor electrodes by combining different nanomaterials and biomaterials, which can detect the threshold concentration by increasing the surface area of the electrode on the basis of the roughness of the electrode. This resulted in the development of new types of sensors such as LSPR and SERS for the amplification of nanoparticle conductivity and spectral power at the nano level. Here, the present review discussed the recent progress of MC biosensors composed of various nanoparticle and bioreceptors. Table 1 showed the recent MC biosensors in terms of nanoparticle types. Thus, the introduction of nanomaterial provides sensitivity and detection method widely. Moreover, those nanomaterials have the different characteristics for MC biosensor construction. Table 2 displays the pros and cons of each nanomaterial in terms of biosensor fabrication. Noble metal-based nanomaterials provide high conductivity, durability, and stability. These characteristics are essential to MC biosensor because MC should be detected in the river or freshwater samples. The high salt, precipitate, and other microorganisms erode the fabricated MC biosensor electrode substrate. However, it is still expensive for manufacturing. The strategy for cost down with noble nanomaterial should be solved for commercialization. The use of carbon-based nanomaterial can be reduced the fabrication cost compared to noble nanomaterial. Moreover, it showed the stable semiconducting property that can bring the chance to fabricate new type of MC biosensor. However, it can be easily oxidized, hampering the sensing performance. Finally, the TMD-based nanomaterial provides the unique conductivity as it called topological insulator; furthermore, the unique chemical property of TMD can provide an opportunity for new concept of MC detection system. However, it still requires a research test for biosensor construction because it usually forms a two-dimensional shape and is hard to particulate.

Although conventional ELISA is mainly based on antibodies and is widely performed to MC detection, the development of aptamers for electrochemical and optical sensors, as well as a new type of ELISA, has become a powerful substitute for antibodies. Aptamers exhibit similar detection capabilities as that of existing antibodies, and because they can be chemically synthesized, they have a low production cost and can be produced more ethically than antibodies. This combination of bioreceptors and nanomaterials can be applied not only for the detection of MC but also to detect other toxic substances such as saxitoxin, anatoxin, and cylindrospermopsin.

However, in order for an actual MC portable sensor to be utilized as a portable biosensor, the following conditions must be met. Although most studies described above were performed on actual samples, most extracted and used only one type of sample to detect toxic substances, that is, blue-green algae. Therefore, an appropriate pretreatment system that can be applied directly in the field should be developed simultaneously. In addition, consistent MC detection ability should be obtained, even for mixed samples such as various blue-green algae or freshwater samples. If miniature equipment is used, the instrument (electrochemical or spectroscopic equipment) performance tends to decrease. Therefore, an optimal size should be considered for MC detection equipment. Finally, the reaction time between the current MC and manufactured bioreceptors/nanomaterial is naturally reversible according to the thermodynamic entropy, but it takes several hours of target binding capacity. Therefore, in order for the detection time to be reduced, an external voltage can be applied to construct an effective portable sensor. If these factors are achieved, an ideal system that can detect the toxic concentration of cyanobacteria directly at the site of occurrence can be developed, rather than conducting a precise analysis in the laboratory.

## Figures and Tables

**Table 1:** MC biosensors composed of various nanomaterials and bioreceptors.

Materials	Bioreceptors	Nanoparticle	Detection Method	Linear Range (μg/L)	LOD (μg/L)	References
Metal-NP	Aptamer	AuNS	CV	0–99.5	9.95 × 10^−4^	[55]
	Antibody	AuNP/PPyMS	LSV	0.25–50	0.1	[56]
	Aptamer	AuNP/MNP	SERS	0.01–200	0.002	[68]
	Aptamer	CuNC	Fluorescence	0.005–1200	0.003	[73]
	Aptamer	AuNP	Fluorescence	0.25–19.90	0.83	[79]
Carbon-NP	Antibody	CNS	DPV	0.05–15	0.016	[85]
	Antibody	CNx-MWNT/AuNP	DPV	0.01–2	0.004	[86]
	Antibody	GO/AuNP	FRET	10^−4^ × 2.5	0.5	[95]
	Antibody	MWCNT	EIS	0.05–20	0.04	[117]
	Antibody	CNF/AuNP	DPV	0.0025–5	0.00168	[118]
TMD-NP	Aptamer	MoS_2_	DPV	0–199	1.99 × 10^−3^	[108]
	Antibody/antibody	MoS_2_/AuNRs	DPV	0.01–20	0.005	[109]
	Aptamer	MoS_2_ QD	Fluorescence	19.90–43.8 × 10^3^	9.95 × 10^−3^	[113]
	Aptamer	MoS_2_	Fluorescence	0.01–50	0.02	[119]
	Antibody/antibody	MoS_2_/AuNCs	DPV	0.001–1000	3 × 10^−4^	[120]
Others	Aptamer	-	Fluorescence	10–100	0.110	[76]
	Aptamer	fluorescence resonance energy transfer based QD	Fluorescence	10^−4^–100	10.4	[114]
	Aptamer	UCNP	Fluorescence	0.1–50	25 × 10^−6^	[115]
	Antibody	Ag@MSN	CA	0.5–30 × 10^3^	0.2	[121]
	Antibody	Cds QD	ECL	0.01–50	0.0028	[122]

**Table 2:** Features and disadvantages of each nanomaterial for MC biosensor construction.

Materials	Features	Disadvantages
Noble metal-based nanomaterial	High stability and durability High conductivity (Ex 1. Ag (6.30 × 10^7^ S/m et 20 °C)) (Ex 2. Au (4.11 × 10^7^ S/m et 20 °C)) [123]	Expensive cost (Ex. Au nanopowder (USD 446 per 1 g))
Carbon-based nanomaterial	Cheap cost Stable semiconducting property (Ex. Carbon nanopowder (USD 11 per 1 g))	Easy to oxidize Low conductivity (Ex 1. carbon-nanotube) (3 × 10^7^ S/m) (Ex 2. graphene (1.72 × 10^7^ S/m)) [124]
Transition metal dichalcogenide-based nanomaterial	Unique conductivity (topological insulator) Unique chemical property	Hard to particulate Low conductivity MoS_2_ (10^−1^ to 10^1^ S/m) [125]

## Data Availability

Not applicable.
